# A facile synthesis of CeO_2_ from the GO@Ce-MOF precursor and its efficient performance in the oxygen evolution reaction

**DOI:** 10.3389/fchem.2022.996560

**Published:** 2022-10-07

**Authors:** Wasif Mahmood Ahmed Malik, Sheereen Afaq, Azhar Mahmood, Li Niu, Muhammad Yousaf ur Rehman, Muhammad Ibrahim, Abrar Mohyuddin, Ashfaq Mahmood Qureshi, Muhammad Naeem Ashiq, Adeel Hussain Chughtai

**Affiliations:** ^1^ Institute of Chemical Sciences, Bahauddin Zakariya University, Multan, Pakistan; ^2^ Department of Chemistry, Emerson University, Multan, Pakistan; ^3^ Guangzhou Key Laboratory of Sensing Materials & Devices, Center for Advanced Analytical Science, School of Chemistry and Chemical Engineering, Guangzhou University, Guangzhou, China; ^4^ Department of Biochemistry, Bahauddin Zakariya University, Multan, Pakistan; ^5^ Department of Chemistry, Government Sadiq College Women University, Bahawalpur, Pakistan

**Keywords:** cerium (3+), MOF (metal–organic framework), GO composites, oxygen evolution reaction, water splitting

## Abstract

Electrochemical water splitting has enticed fascinating consideration as a key conduit for the advancement of renewable energy systems. Fabricating adequate electrocatalysts for water splitting is fervently preferred to curtail their overpotentials and hasten practical utilizations. In this work, a series of Ce-MOF, GO@Ce-MOF, calcinated Ce-MOF, and calcinated GO@Ce-MOF were synthesized and used as high-proficient electrocatalysts for the oxygen evolution reaction. The physicochemical characteristics of the prepared samples were measured by diverse analytical techniques including SEM, HRTEM, FTIR, BET, XPS, XRD, and EDX. All materials underwent cyclic voltammetry tests and were evaluated by electrochemical impedance spectroscopy and oxygen evolution reaction. Ce-MOF, GO@Ce-MOF, calcinated Ce-MOF, and calcinated GO@Ce-MOF have remarkable properties such as enhanced specific surface area, improved catalytic performance, and outstanding permanency in the alkaline solution (KOH). These factors upsurge ECSA and intensify the OER performance of the prepared materials. More exposed surface active-sites present in calcinated GO@Ce-MOF could be the logic for superior electrocatalytic activity. Chronoamperometry of the catalyst for 15°h divulges long-term stability of Ce-MOF during OER. Impedance measurements indicate higher conductivity of synthesized catalysts, facilitating the charge transfer reaction during electrochemical water splitting. This study will open up a new itinerary for conspiring highly ordered MOF-based surface active resources for distinct electrochemical energy applications.

## Introduction

Metal–organic frameworks (MOFs) and metal–organic polyhedras (MOPs) have collectively enticed a matchless attraction in research fields all around the world (Chughtai et al., 2015). Being an advanced class of porous materials, MOFs have unified characteristics of both microporous as well as mesoporous materials inside a single framework, that is, incomparable flexibility (Hou et al., 2020), exceptional feature of permanent porosity (Zhang et al., 2020), highly crystalline (Amaro-Gahete et al., 2019), uniform pore sizes (Wu et al., 2021a), stable network (Baburin et al., 2008), uniform and controllable morphologies (Nandiyanto and Okuyama, 2011), excellent thermal stability (Liang et al., 2022), exceedingly larger surface areas (Farha et al., 2012) along with a unique feature of pore size tunability (Bonnett et al., 2020), improved pore dimensionality (98°Å) (Deng et al., 2012) with the surplus edge of lower density, that is, 0.13°g cm^−3^ (Furukawa et al., 2011), and versatile host–guest interactivity (Gao et al., 2021) and functionality (Kim and Hong, 2021).

MOFs and their derivatives are capable of getting modernized readily for their respective use in particular applications entailing gas storage (Chaemchuen et al., 2013), molecular separation (Petit, 2018), sensing (Lustig et al., 2017), adsorption (Wu et al., 2021b), heterogeneous catalysis (Chughtai et al., 2015), solar cells (Fang et al., 2018), drug delivery (Cao et al., 2020), luminescence (Allendorf et al., 2009), electrode materials (Wu et al., 2019), carriers for nanomaterials (Nadeem et al., 2018), magnetism (Thorarinsdottir and Harris, 2020), polymerization (Goetjen et al., 2020), imaging (Bieniek et al., 2021), membrane (Zornoza et al., 2013), suitable for storage of fuels (Nik Zaiman et al., 2022), thin-film systems (Shekhah et al., 2011), proton conduction (Shimizu et al., 2013), renewable energy (Zhang et al., 2017a), capture of CO_2_ (Ding et al., 2019), and therapeutics and diagnostics in biomedicine (Liang et al., 2015; Bara et al., 2019; Falahati et al., 2022).

Ever-increasing world population, diminishing fossil fuel reserves, and prompt industrial development have compelled the global power sector thrive to find out an alternate for sustainable energy reservoirs. It is estimated that by 2050 the overall population will witness an escalation of 26%, that is, roughly 9.7 billion, that would result in approximately 50% rise in overall world’s primary energy consumption (Conti et al., 2016; Gu et al., 2021). Natural energy sources are being consumed in a licentious routine.

According to a survey, if global warming prolongs at its current percentage, then the global temperature is expected to upturn by 1.5°C within a time span of 2030–2052, and a consequential rise of 3.8°m in the sea level would be experienced (Waisman et al., 2019). To take up arms against these problems, a substantial concern for exploring clean as well as renewable substitute of energy resources is mandatory for succeeding a viable future accordingly (Shi and Zhang, 2016). The accomplishment of essential goals is the main focus of clean energy, that is, 1) enriched resources consumption, 2) boosted proficiency, 3) greener ecosystem, 4) economic compatibility, 5) upgraded energy sanctuary, and 6) effectual study and designing (Dincer and Acar, 2015).

One of the most proficient solutions regarding clean energy benefaction is hydrogen to be used as a clean energy substitute, as it is the most proficient and viable green practice. Additionally, garnered solar energy is capable enough to meet our energy consumption demands only by means of solar energy cells as well as *via* accomplishment of the water-splitting reaction occurring on behalf of photo-electrocatalytic pathways that tend to use sunlight for the conversion of water into hydrogen. Having inestimable potential, hydrogen is taken as an uplifting substitute for energy reserves.

Hydrogen has a nontoxic nature with large combustion efficacy and gives out clean exhaust products. Having a renewable nature, hydrogen is taken as a plentiful reservoir of storable clean energy fuel as its energy density (120°MJ/kg) is comparatively higher than that of gasoline (44°MJ/kg) (Dincer and Acar, 2015; Acar and Dincer, 2019). Though cost-effective hydrogen is anticipated to be supremely preferential sustainable energy resolution economically as well, for its profitable applications, still most of the hydrogen production practices are in a progressing phase.

Among all the renewable substitutes adapted for the production of hydrogen, water-splitting is regarded as a conspicuous method (Fajrina and Tahir, 2019). The most abundant resource on Earth is water as water covers 70% of the total Earth’s surface. The water-splitting process consumes water as a feedstock and is definitely liable to be recycled back into the ecosystem, hence, tends to produce hydrogen with a virtually zero environmental impact. Oxygen is a side product of this process, so no obvious environmental pollution is conceived, and the side product is used for carrying out other relevant processes that directly or indirectly enhances the commercial cost-effectiveness of overall electrolysis practice. Currently, water electrolysis carries out only 4% of the overall hydrogen production globally. Higher energy consumption for breaking the hydrogen bonding prevailed in a water molecule along with considerable investment are resistive forces used for carrying out hydrogen production *via* water electrolysis.

A two-half-cell reaction strategy is employed for water-splitting, that is, 1) OER (oxygen evolution reaction) and 2) HER (hydrogen evolution reaction). Although reaction kinetics for both reactions is a sluggish one, there is an utmost need of use of a catalyst for overpowering the strong chemical bonds persisting in hydrogen and oxygen atoms of water molecules for completing conversion in an auspicious manner. Usually, the noble metal-based catalysts are employed in water-splitting, that is, Ir/Ru- and Pt-based catalysts. The disadvantages of the aforementioned catalysts entail economic factors, limited reserves, and poor stability which limits the large-scale practices for the electrolysis process (Rosen, 2010). Alternatively, owning to their easy accessibility as well as extraordinary activity with the edge of enhanced stability, the abundantly found transition metals, for example, Co, Fe, and Ni, can be better substitutes for noble metal-based catalysts (Anantharaj et al., 2016). Additionally, catalyst morphology and make-up procedures play an integral role for determining the catalytic action of the water-splitting catalyst. Consequently, for effectual hydrogen production, the catalyst material’s optimization as well as morphological features has vital prominence.

In previous years, the application of MOF edifices as electrocatalysts as well as photo-electrocatalysts in water-splitting is being studied immensely. Attentiveness in this field of research will endure intensification as well. The intrinsic property of MOF makes them capable enough to retain structural features and to sustain their functionality. Additionally, for water-splitting, the post-synthetic reforms mark them as fascinating catalyst material (Wang et al., 2017). MOF edifices offer astonishing electronic, spatial, chemical, and physical flexibility for supporting and sustaining the water-splitting reactions accordingly. For water electrolysis perception, MOF edifices can be designed for operative and boosted catalytic sites, familiarizing the light sensitization and tunability of band gap along with photo-generated charge sustainability. MOF edifices hold certain distinctive advantages when compared with conventional semiconductor catalysts and hence can be employed as templates that are capable of serving as precursors for functionalized material synthesis, for example, carbon metal/metal oxide hybrid synthesis, porous carbon synthesis, metal sulfide synthesis, and metal oxide synthesis (Sun et al., 2017). In recent times, MOF edifices are also being employed as precursors for nanometal oxides or nanoporous carbon synthesis (Hasan et al., 2016). Subsequent products mostly preserve the structural morphologies inherited from their parent MOF templates and hence present an enhanced catalytic activity in many cases (Su et al., 2017). For the fabrication of highly ordered nanocatalysts, MOF edifices and their derivatives can also be engaged as supports as well. MOF edifices that have prospective facilitate the exactitudes of water splitting enlist a number of PCN (Zr-porphyrins), SIM-1 (Zn), MIL-101 (Al and Cr), ZIF-8 (Zn), UiO-66 (Zr), MIL-125(Ti), MIL-100 (Cr), and UiO-67(Zr).

By targeting both benefits inside the single catalyst entity, that is, enhancing conductivity ratio *via* functionalization and rising the number of active sites can result in superior electrochemical performance of water splitting. Furthermore, during pyrolysis, the atomic-level as well as molecular-level rearrangement is only allowed by the flexible MOF-based skeleton. Correspondingly, for the thermal synthesis of porous MOF-derived edifices and carbon-based nanomaterials (including metals, single-atom catalysts (SACs), and metal compounds), respective MOFs or relevant MOF-based composites serve as templates equally. The preplanned pyrolysis of MOFs provided with highly ordered calcination modifies a number of characteristics including porosity, conductivity, surface area, catalytic performance, and stability. Therefore, such derivatives are of great interest for the water-splitting purpose. Among these approaches, combining MOFs with suitable materials for synthesizing their composites is one of the liable techniques. The materials to be inserted in MOF edifices include graphene-based materials, metals, porous carbon (Qian et al., 2012), and carbon nanotubes (CNTs) (Xiang et al., 2011) (Lim et al., 2012) (Zhu and Xu, 2014). Recently, the latter substrate has multiplied pronounced dominance. Usually, insertion of any selected conductive functional entity inside MOF edifices has anticipated being an operative technique for enlightening their electrochemical presentation (Hosseini et al., 2013; Zhang et al., 2019a).

Carbon material having a single-atom thickness, namely, graphene, possesses a number of advantageous capabilities such as outstanding electronic conduction, chemical robustness, amenableness, and enhanced surface area property that marks them as perfect mechanical supportive templates for respective MOF materials (Xia et al., 2016). Since the last decade, a great attention has been engrossed by graphene in both experimental as well as theoretical scientific fields. For the reason that it possesses specific exceptional electronic, structural, thermal and mechanical and properties (Zhu et al., 2010), the graphene and its derivatives have found widespread applications as innovative carbon nanomaterial in innumerable fields, enlisting chemical and electrochemical sensors, electronic devices, energy storage, and catalysis in biological as well as biomedicine applications (Jiang, 2011; Xiang et al., 2012; Dai, 2013; Wu et al., 2013).

Attractive support material, namely, graphene oxide (GO) possesses chemical stability, large specific surface area, ease of accessibility, edge reactivity, electrical conductivity, facile synthesis, and exceptional mechanical properties (Wang et al., 2010). Owing to the presence of GO, a unique 2D lamellar edifice is possessed by the derivatives of graphene (Dreyer et al., 2010). For synthesizing graphene-based soft materials and relevant hybrid composites, the functionalized basal plane of GO having anchoring moieties like carboxyl, hydroxy, or epoxy groups is advantageous (Kim et al., 2012; Anantharaj et al., 2016). The dispersibility feature of MOF and adsorption of small molecules are facilitated by GO, as it possesses comparatively higher atomic density along with the presence of a large amount of oxygen-based moieties exposed on to the surface thoroughly (Petit et al., 2011; Pei et al., 2018).

For fabrication of composite materials, the aforementioned functional nature of the GO surface marks it as one of the excellent nanoscale building units having suitable reaction sites in it (Kumar et al., 2014). The central positively charged constituent of the MOF edifice interacts with that entity of GO which possesses oxygen, and hence synthesis of a hybrid complex is accomplished. The combination of GO and MOF edifices would fashion additional micropores at the interacting edges of both constituents that will result in improved accessible sites for reactants. These newly created sites would serve as active sites and will definitely moderate the mass-transfer-limitation effects that were hindering the catalysis process (Zubir et al., 2015). Additionally, prior studies also revealed that GO is capable to drive the internal electron transfer process inside the composite edifice and is also capable for inducing a synergistic effect stuck between both constituents for improving the catalytic performances (Jahan et al., 2010).

For the synthesis of new multifunctional composites/hybrids, the tractable incorporation of MOFs and functional materials has proved to be a leading-edge, and the resultant hybrids unveil new capabilities that have proven to be superior to the capabilities of individual constituents achieved by the cooperative interaction of the functional entities (Rodenas et al., 2015). Enduring efforts have been dedicated for the synthesis of graphene/GO-MOF composites for assembling the exclusive properties of both individual constituents, that is, graphene/GO layers and MOF edifices for relevant applications. During the last 3°years, plentiful efforts were made for synthesizing GOMOF composites, for instance, GO-MIL-53 (Zhang et al., 2014), GO-HKUST-1 (Petit et al., 2011), GO-ZIF-8 (Kumar et al., 2013), GO-MOF-5 (Jahan et al., 2010), GO-ZIF-67 (Wei et al., 2015), and GO-MIL-100 (Petit and Bandosz, 2011), that aimed the assembly of individual distinctive features of major constituents, namely, MOFs and GO layers that will further make their virtual practical implementation possible in the desired field for specified applications. In the aforementioned technique, precursors for MOF preparation, namely, the metal salt and selected organic linker moieties are combined with presynthesized constituting materials in relevant suitable solvents characteristically engaged for pure MOF synthesis. At that point, the synthesis practice proceeds on behalf of conventional electric heating of the reaction mixture under ambient reaction conditions (i.e., duration and temperature) that are usually provided for the synthesis of pure MOFs (Stock and Biswas, 2012).

Excellent redox capability of cerium could be attributed to its ability of valence state transition, that is, from Ce^4+^ to Ce^3+^. Hence, cerium (a member of rare earth metals) is extensively being used for the catalysis purpose (Levin et al., 2016). Additionally, MOF edifices possess captivating properties, that is, outstanding porosity and enhanced specific surface area accordingly. In electrochemical catalysis, these features make the Ce-MOF-based composite an auspicious material so far.

Perceiving the prosperity of GO, cerium, and MOFs, we synthesized a MOF–graphene oxide composite (GO@Ce-MOF) by an *in situ* growth method. Graphene oxide not only has virtuous conductivity but also functions as a pillar for linking MOF nodes, which upsurge stability of composite materials. It is generally anticipated that more new pore spaces will be assembled at the interface between GO and MOF blocks, which can upturn dispersive forces and can adequately hold guest molecules for catalysis. Finally, pure CeO_2_ have been efficaciously synthesized by pyrolysis of Ce-MOF and its composite which retain the mesoporous structure. Ce-MOF, its composite (GO@Ce-MOF), calcinated Ce-MOF, and calcinated GO@Ce-MOF underwent cyclic voltammetry tests and were evaluated by electrochemical impedance spectroscopy and oxygen evolution reaction. The catalytic recital of CeO_2_ has been expansively studied by equating with Ce-MOF and GO@Ce-MOF. It is found that Ce-based materials show the best catalytic performance in electrochemical impedance spectroscopy and oxygen evolution reaction. These stable, economical, and adequate electrocatalysts are expected to be pre-eminent electrocatalysts for OER.

## Materials and methods

All chemicals used in this project were purchased from Sigma-Aldrich. The chemicals were utilized as such, however, when necessitated, were purified by common techniques, that is, recrystallization and distillation. FTIR spectra were traced on the PerkinElmer Spectrum One spectrometer. By exploiting the Vario EL cube, elemental analyses were obtained. Using the Empyrean instrument from PANalytical, the PXRD (X-ray powder diffraction) patterns were collected by engaging monochromatic Cu Kα under ambient conditions. SEM (scanning electron microscopy) images were executed on the JEOL (0.5–35°KV, JSM-5610LV). HRTEM (transmission electron microscopy) studies were performed using the Philips CM20 microscope functioned at 200°KV. On behalf of the PerkinElmer PHI (5000C ESCA), the XPS (X-ray photoelectron spectroscopy) analysis was completed. With the help of N_2_ adsorption/desorption isotherms collected on to the Micrometrics instrument (ASAP 2020) at 77°K temperature, the pore size and BET surface area measurements were perceived. Although prior to BET analysis, the prepared materials were thoroughly degassed overnight by providing 150 °C temperature.

### Preparation of Ce-MOF

**SCHEME 1:**
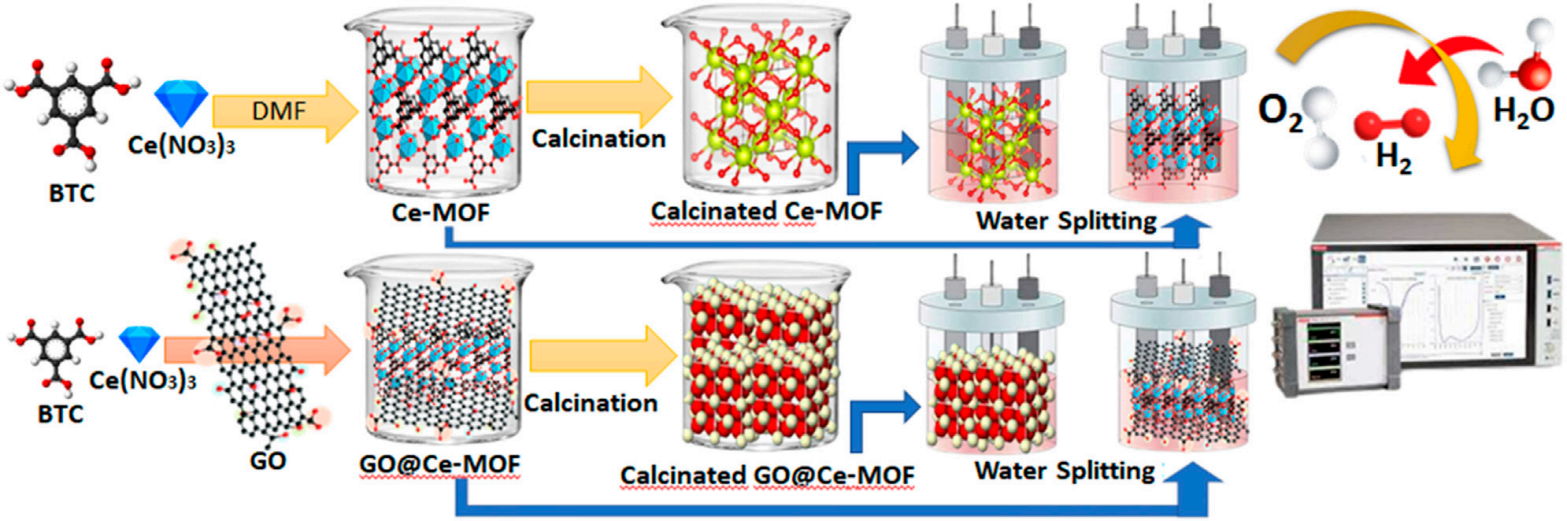
Schematic of Ce-MOF, GO@Ce-MOF, calcinated Ce-MOF, and calcinated GO@Ce-MOF and their OER study.

The reaction mixture containing 10°mmol Ce(NO_3_)_3_.6H_2_O, 6°mmol 1,3,5-benzenetricorboxylic acid (BTC), and 60°ml of DMF was stirred for 15°min. The said mixture was placed inside the Teflon that was placed inside a stainless steel autoclave and kept at 130°C for 24°h. After 24°h, this autoclave was cooled thoroughly until room temperature was established. The prepared colorless crystals were centrifuged and collected after washing with DMF (Scheme 1). The collected material was then dried at 60°C and then packed and stored for further investigations and applications.

### Preparation of Ce-MOF composite with graphene oxide

A homogenous mixture of 10.00°mmol Ce(NO_3_)_3_.6H_2_O, 6.00°mmol 1,3,5-benzenetricorboxylic acid (BTC), and 60°ml of DMF was added with a suspension of 1°g graphene oxide (GO) and stirred for 15°min. The said mixture was placed inside the Teflon that was placed inside a stainless steel autoclave and kept at 130°C for 24°h. After 24°h, this autoclave was cooled thoroughly until room temperature was established. The prepared dark brown crystals were subjected to centrifugation and were collected after consequently washed with DMF (Scheme 1). The collected material was then dried at 60°C and then packed and stored for further investigations and applications.

### Calcination of graphene oxide composite of MOF

Solvothermally synthesized composites of cerium-based metal–organic frameworks with graphene oxide were further subjected to calcination at 500°C for 5°h. Subsequently, the resultant product was finely ground and collected in a vacuum desiccator and then was stored for subsequent use.

### Preparation of the working electrode

To find the application of the material synthesized for better electrochemical performance, working electrode preparation is the key step. For this purpose, 15°mg of the synthesized MOF was dispersed in 100°µL volume of deionized water, and this suspension was kept for sonication for 1°h. After sonication, the working electrode ink was prepared. The glassware was washed thoroughly for reuse. The identical procedure was repeated for GO@Ce-MOF, calcinated Ce-MOF, and calcinated GO@Ce-MOF.

The prepared ink was deposited on the nickel foam (NF) substrate which was cut into 1 × 1°cm dimension. Before this deposition, NF pieces were thoroughly washed with acetone, then with 2°M hydrochloric acid followed by deionized water, and ending up in washing with ethanol; 20-min washing was completed for each solvent individually. After washing with the aforementioned solvents in the respective order, the washed NF pieces were placed for drying at 60°C, and the prepared slurry of Ce-MOFs was applied on NF pieces by taking 10°µL in the micropipette. Overnight drying of NF with the sample was accomplished at room temperature, and then it was used as the working electrode for the catalysis study. Similarly, the composites (GO@Ce-MOF), calcinated Ce-MOFs, and calcinated GO@Ce-MOF were applied on the NF substrate.

## Results and discussion

### FTIR

**FIGURE 1:**
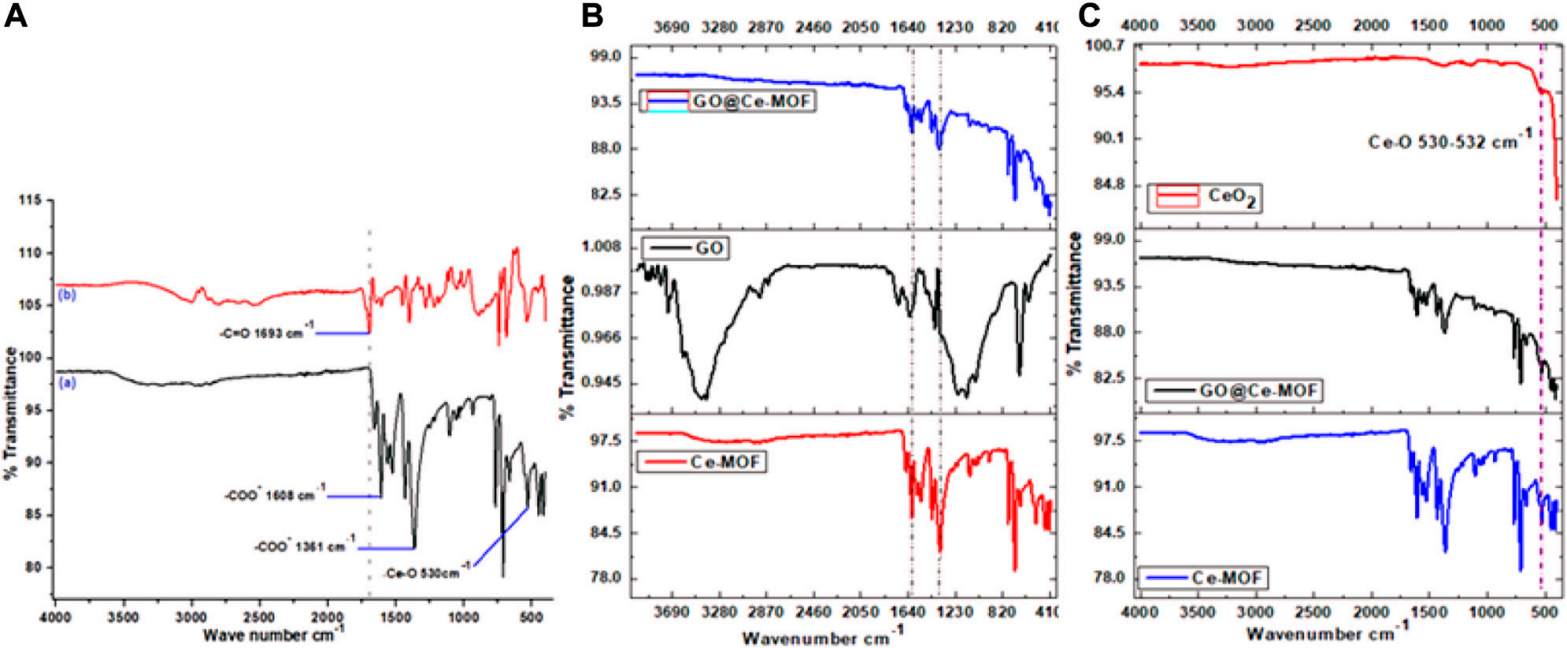
**(A)** FTIR of BTC linker (b) and Ce-MOF, **(B)** FTIR of Ce-MOF, GO, and GO@Ce- MOF, and **(C)** FTIR of Ce-MOF, GO@Ce-MOF, and calcinated GO@Ce-MOF (CeO_2_).

FTIR spectra of Ce-MOF and BTC (organic linker) are shown in Figure 1. The FTIR spectra of mesoporous Ce-MOF revealed the presence of two important peaks at 1,361 and 1,608°cm^−1^ that describe the vibrations of anionic carboxylate moieties (υ-COO^**-**^ 1,630–1,500°cm^−1^) and antisymmetric and symmetric stretching (υ-COO^−^ 1,400–1,310°cm^−1^), which were correlated to anionic carboxylate moieties’ stretching vibrations and were found to be lower than the value of υ (C=O) stretching vibration (1760–1,690°cm^−1^) perceived for free carboxylic acids (Figure 1A). The two aforementioned peaks were correlated with the carboxylate ions’ stretching vibrations. This confirms the presence of carboxylate ions inside the material to be examined. The strong absorption bands of the carbonyl group utterly disappeared at 1,693°cm^−1^ (that is, correlated with the O-donor carboxyl ligand moiety) that pointed out full deprotonation of carboxylic acid groups in Ce-MOF consequently, transformation of carboxyl moieties into carboxylate ones, and also confirmed coordination between Ce and carboxylate group of the organic linker upon the reaction with metal ions. The coordination of Ce^3+^ ions with the provided ligand, namely, 1,3,5-H_3_BTC, resulting in successful synthesis of Ce-MOF edifice that can be demonstrated by these bands. For the BTC ligand, the aromatic ring-based C = C vibrations are represented by the absorption bands at frequencies of 1,563, 1,558, and 1,525°cm^−1^. At 1,431°cm^−1^ (C = C 1500–1,400), the C-C stretching (in-ring) emerges. The in-plane bending ( = C-H) was observed at 1,104°cm^−1^, while out-of-plane bending ( = C-H) was observed at 709°cm and 768°cm^−1^. Additionally, three deformation vibrations were also noted for BTC (Almáši et al., 2015).

At 2,937 and 2,867°cm^−1^, the aliphatic C-H groups of DMF exhibit too weak stretching vibrations for asymmetric and symmetric observations, respectively. Moreover, the presence of organic solvent (coordinated DMF) is evident from the medium band at 1,657°cm^−1^ for Ce-MOF, which belongs to the tensile stretching vibration mode for the carbonyl group (C = O) of coordinated DMF ligands, indicating that strongly adsorbed solvent molecules tend to stabilize the framework of prepared MOF (Figure 1A). The coordination with the Ce metal ion is what causes the characteristic peak to shift slightly toward the lower wavenumber area when compared to the value of free DMF molecules (1,667°cm^1^) (Choi et al., 2016). The C-O stretching vibration in BTC was observed at 1,400°cm^−1^. It was found that in the spectra of Ce (BTC) that this peak was declined to 1,359°cm^−1^, authenticating oxygen coordination with that of metal ions (Ce^3+^). It is noticeable that a peak appeared at 530°cm^−1^ wavelength was resulted by Ce-O bond’s stretching vibration and proved the successful coordination of Ce^3+^ ions with oxygen groups of the organic linker, which was seen in the range of 500–650°cm^−1^ (Maiti et al., 2014). The coordination of the organic ligand, namely, 1,3,5-H_3_BTC with that of metal salt of Ce resulted in the effective preparation of Ce-MOF is shown by each of these peaks.

According to standard reports for GO, the FTIR spectrum of GO showed major stretching vibrations at 3,433 and 3,394°cm^−1^ (-O-H stretching), 2,929 and 2,857°cm^−1^ (CH_3_ and CH_2_ methylene groups stretching), 1,723°cm^−1^ (-C = O stretching in carbonyl or carboxyl groups), 1,628°cm^−1^ (C = C stretching), 1,204 and 1,131°cm^−1^ (C-O stretching), and 1,050°cm^−1^ (C-O-C stretching) (Figure 1B). These provide a proof that GO has several oxygenous functional groups. These GO absorption peaks demonstrate the effective synthesis of thin GO nanosheets using graphite as a precursor (Yang et al., 2020).

The successful synthesis of GO@Ce-MOF composite was strongly evidenced by FT-IR spectra presented in Figure 1B. The spectrum of GO@Ce-MOF was analogous to Ce-MOF′ spectra. Hence, the presence of BTC inside the hybrid composites, that is, GO@Ce-MOF was assured (Bandosz and Petit, 2011; Ma et al., 2017). The Go@Ce-MOF composites could be observed to have the same pattern of Ce-MOF characteristic bands, indicating that Ce-MOF has been efficaciously grafted onto the GO’s surface. The characteristic absorption bands of oxygen-containing groups of GO, for e.g., 3,343 and 3,394°cm^−1^ (O–H stretch), 1,723°cm^−1^ (C = O stretch), 1,204 and 1,131°cm^−1^ (C-O stretching), and 1,050 (C-O-C stretching) are not witnessed in the GO@Ce-MOF’s spectrum. The possible reason for this fact is that they got coordinated with metallic cations, that is, Ce^3+^. This may be because the GO layer containing oxygen functional groups are connected on the open metal sites of Ce-MOF composite, and thus these bands for GO disappear (Figure 1B). (Cao et al., 2015).

In addition, the analogous FT-IR spectra for Ce-MOF and GO@Ce-MOF illustrate that a large amount of GO assists to organize the edifice of Ce-MOF crystals in an efficient way. This outcome verifies the supportive role of GO that tends to serve as a template to fasten the Ce^3+^ cations that additionally facilitate the GO@Ce-MOF crystals to grow onto its surface, without destroying the basic structure of the coordination framework of Ce-MOF accordingly. It is shown that all the GO@Ce-MOF composites exhibit similar bands as those in the parent Ce-MOF, indicating that the incorporation of graphene oxide did not prevent the formation of Ce-MOF. After the calcination at temperature 500°C, the characteristic peaks for GO@Ce-MOF and for Ce-MOF completely disappeared in the calcinated GO@Ce-MOF FTIR spectrum, and the final product is identified as CeO_2_. Additionally, it can be deduced that following calcination, the GO@Ce-distinctive MOF’s organic solvent DMF peaks were also virtually entirely eliminated. The stretching frequency of Ce-O in the metal oxide network CeO_2_ is what causes the band below 700°cm^−1^ to exist (Zawadzki, 2008) and appears in the case of Ce-MOF and also in cases of GO@Ce-MOF and calcinated GO@Ce-MOF. The characteristic peak for the Ce-O stretching vibration was evident in the form of an intense band at approximately 530–532°cm^−1^ (Figure 1C).

### PXRD

**FIGURE 2:**
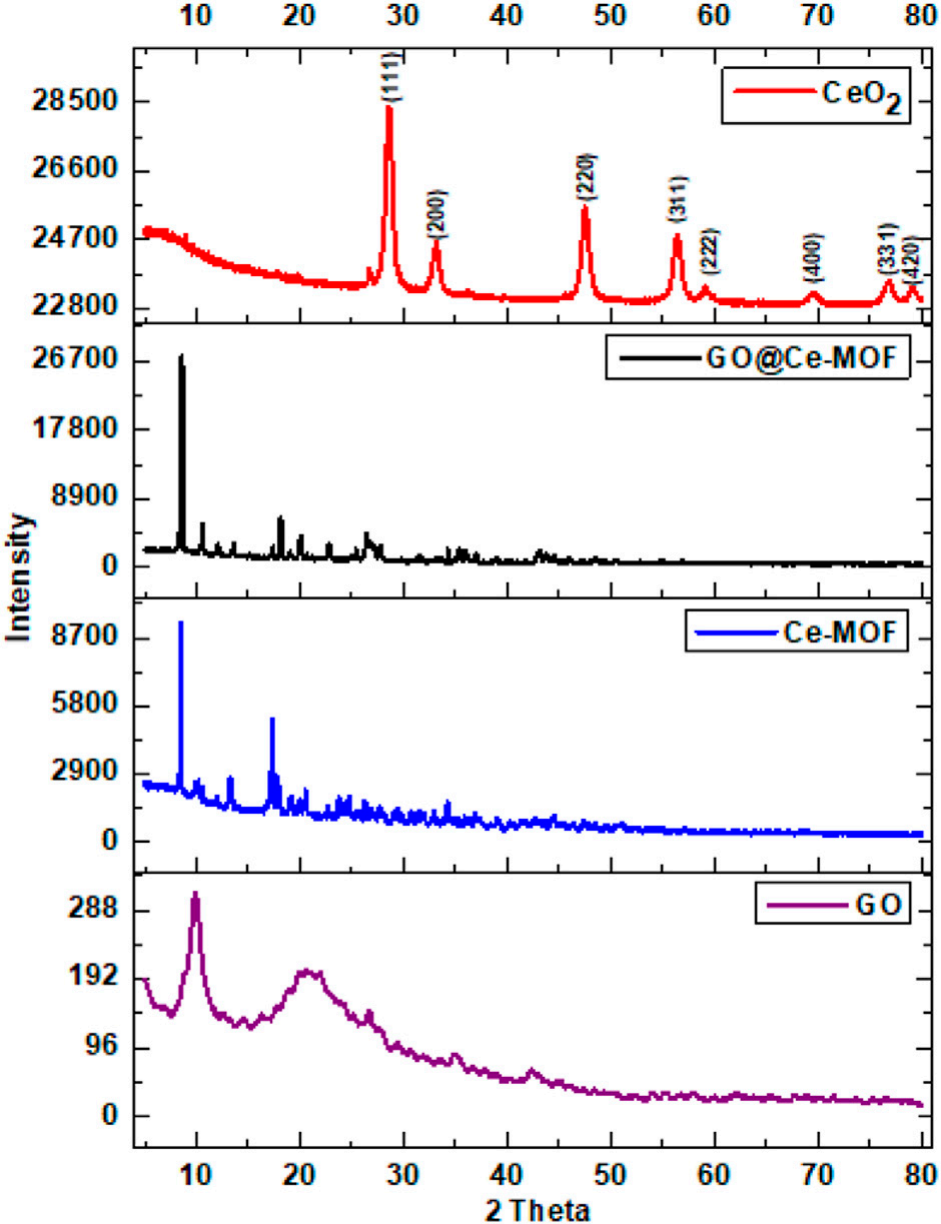
Powder X-ray diffraction patterns of GO, Ce-MOF, GO@Ce-MOF, and CeO_2_.

Powder X-ray diffraction was utilized to examine the phase purity and crystallinity of the prepared materials, namely, Ce-MOF, calcinated GO@Ce-MOF or (CeO_2_), and GO@Ce-MOF (composite). Figure 2 illustrates the XRD patterns obtained for Ce-MOF and also for calcinated Ce-MOF.

The XRD pattern of Ce-MOF shows the characteristic peaks at (2θ) 8.5, 17.3, 20.5, 24.7, and 34.2. Ce-MOF has a fine structure and high degree of crystallinity as evidenced by the sharpness and clarity of each of its diffraction peaks (Figure 2). The XRD pattern that has been previously described in the literature and all of the diffraction peaks are in excellent agreement (Zhang et al., 2017b; Lin et al., 2017; Zhang et al., 2019b). The PXRD pattern corresponded to that of another family of Ln (BTC) MOFs. Each BTC linker is coordinated to six Ce^3+^ ions, and each carboxylate group linker is coordinated to two different Ce^3+^ ions. The inorganic building unit is based on distorted pentagonal CeO_7_-bipyramids in which six oxygen atoms form a part of carboxylate groups, while one oxygen atom belongs to a solvent molecule, water, or DMF. This wriggling in and out of the plane in two directions arranges the CeO_7_-units into helical chains forming 43-screw axes, leading to the formation of noninterconnected square-shaped channels (Ethiraj et al., 2016). Additionally, there are no peaks connected to any impurities, indicating that the sample has a high phase purity. GO shows a prominent peak at about 9.89°, which denotes an interlayer distance of about 9.5. Comparing the GO surface to pure graphite, the increase in interlayer spacing from 0.335° (2 theta = 26.6) to 0.836°nm demonstrated the presence of oxygen functions.

Peaks having two theta values of 8.5, 10.6, 18.1, 26.3, and 34.2° are seen in the XRD spectrum of GO@Ce-MOF. In GO@Ce-MOF, the XRD pattern entails each and every peak that was observed for Ce-MOF; however, the peak at two theta of 12, which is indicative of the GO structure, almost does not appear. This could be the possible explanation for the prospect of GO@Ce-MOF crystals being evenly distributed throughout the spaces between GO sheets. The characteristic peaks of Ce-MOF and its MOF-76 family may be seen in a diffractogram of GO@Ce-MOF at 8.4 and 10.6° (Almáši et al., 2015; Almáši et al., 2016; Zhang et al., 2017b; Zhang et al., 2018; Santos and Luz, 2020), proving that GO in place has no effect on the synthesis of Ce-MOF. The synthesized Ce-MOF shows highly ordered Ce-MOF units inside the respective composite, and the diffraction peaks of Go@Ce-MOF indicate that GO’s presence obstructing neither the crystallization of the Ce-MOF edifice nor that of the composites having a Ce-MOF structure in it.

By using XRD, as shown in Figure 2, the analysis of crystallinity along with total crystal phases of the synthesized CeO_2_ was accomplished. At 2θ = 28.5, 33.1, 47.5, 56.5, 59.1, 69.3, 76.8, and 79.1° that can be correlated with (111), (200), (220), (311), (222), (400), (331), and (420) planes, correspondingly, PXRD showed well-defined peaks. Only CeO_2_ is the source of all obtained peaks in the pattern. All of these synthetic CeO_2_ peak heights are inter-related with the face-centered cubic phase of the compound (JCPDF cards no. 75-8371) (Khan et al., 2011). The peak patterns of Ce-MOF and GO@Ce-MOF have completely distinct XRD patterns, which point to CeO_2_ being produced when both GO@Ce-MOF and Ce-MOF are pyrolyzed. The PDF cards indicate that the lattice parameter for CeO_2_ particles is 5.4116. Only CeO_2_ is the source of all obtained peaks in the pattern. No further impurity-related signal was seen, indicating that the produced catalyst is pure CeO_2_ with a cubic phase (Niu et al., 2009).

### Morphological characterization

#### SEM

**FIGURE 3:**
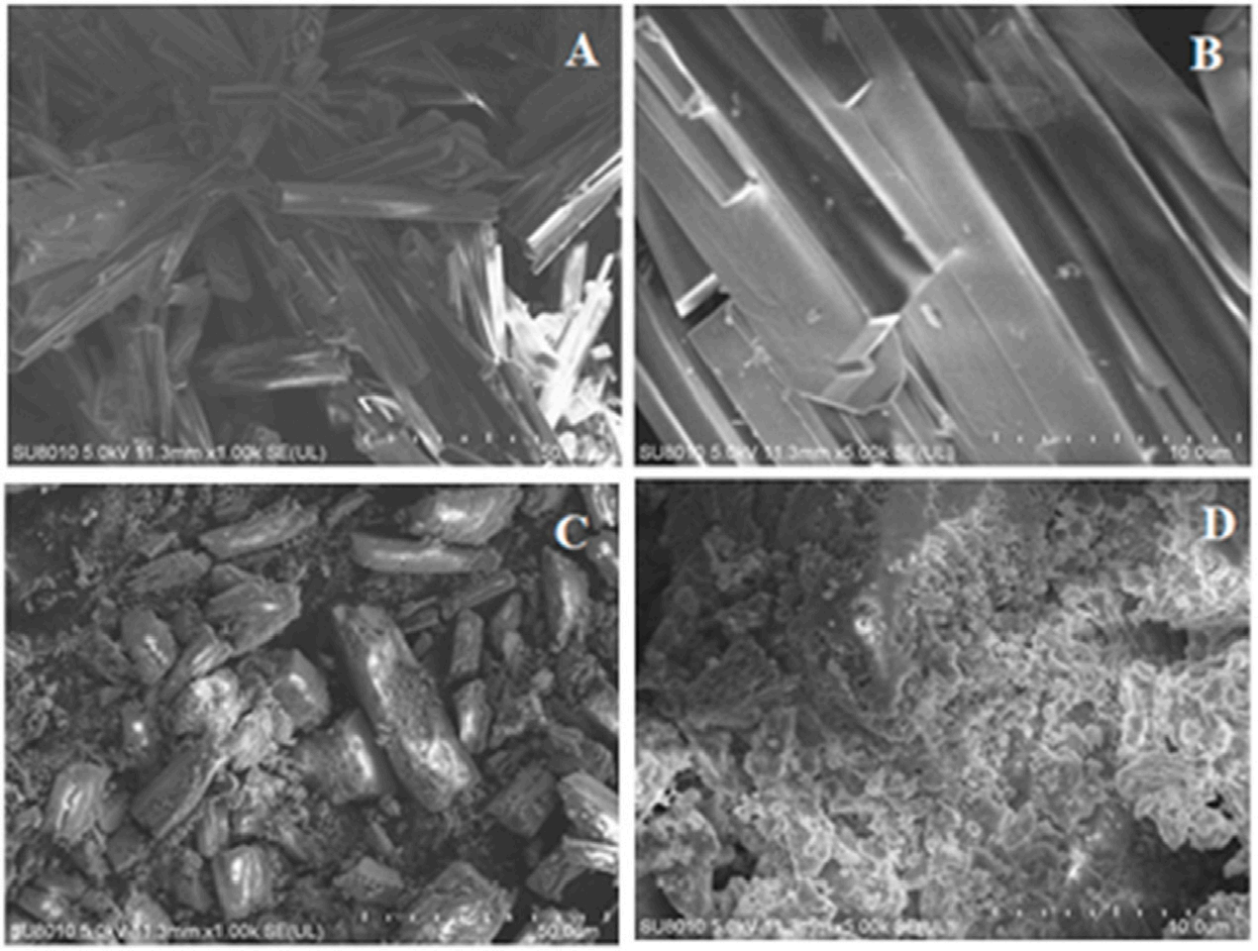
SEM images for **(A,B)** Ce-MOF and **(C,D)** GO@Ce-MOF composite.

The morphology of as-synthesized materials, namely, Ce-MOF and GO@Ce-MOF composite was characterized using high-resolution transmission electron microscopic (HRTEM) analysis and scanning electron microscopic (SEM) analysis. Furthermore, the elemental composition of every single element was studied by energy-dispersive spectroscopy (EDS). Figures 3A,B illustrations were taken at two altered magnifications for SEM descriptions of Ce-MOF. The rod-like morphology of Ce-MOF was clearly visible, indicating the successful synthesis of MOFs from BTC linkers. On the other hand, SEM analysis for GO@Ce-MOF composite is illustrated in Figures 3C,D. Graphene oxide is attached exteriorly on to the rod-like surface of prepared Ce-MOFs that confirms the formation of GO@Ce-MOF composite evidently. There is a clear difference in the morphology of pure Ce-MOF and its corresponding composites. These results suggest the successful formation of Ce-MOF and its mentioned respective composites on behalf of graphene oxide in addition to graphene oxide quantum dot composites accordingly.

#### HRTEM and EDS

**FIGURE 4:**
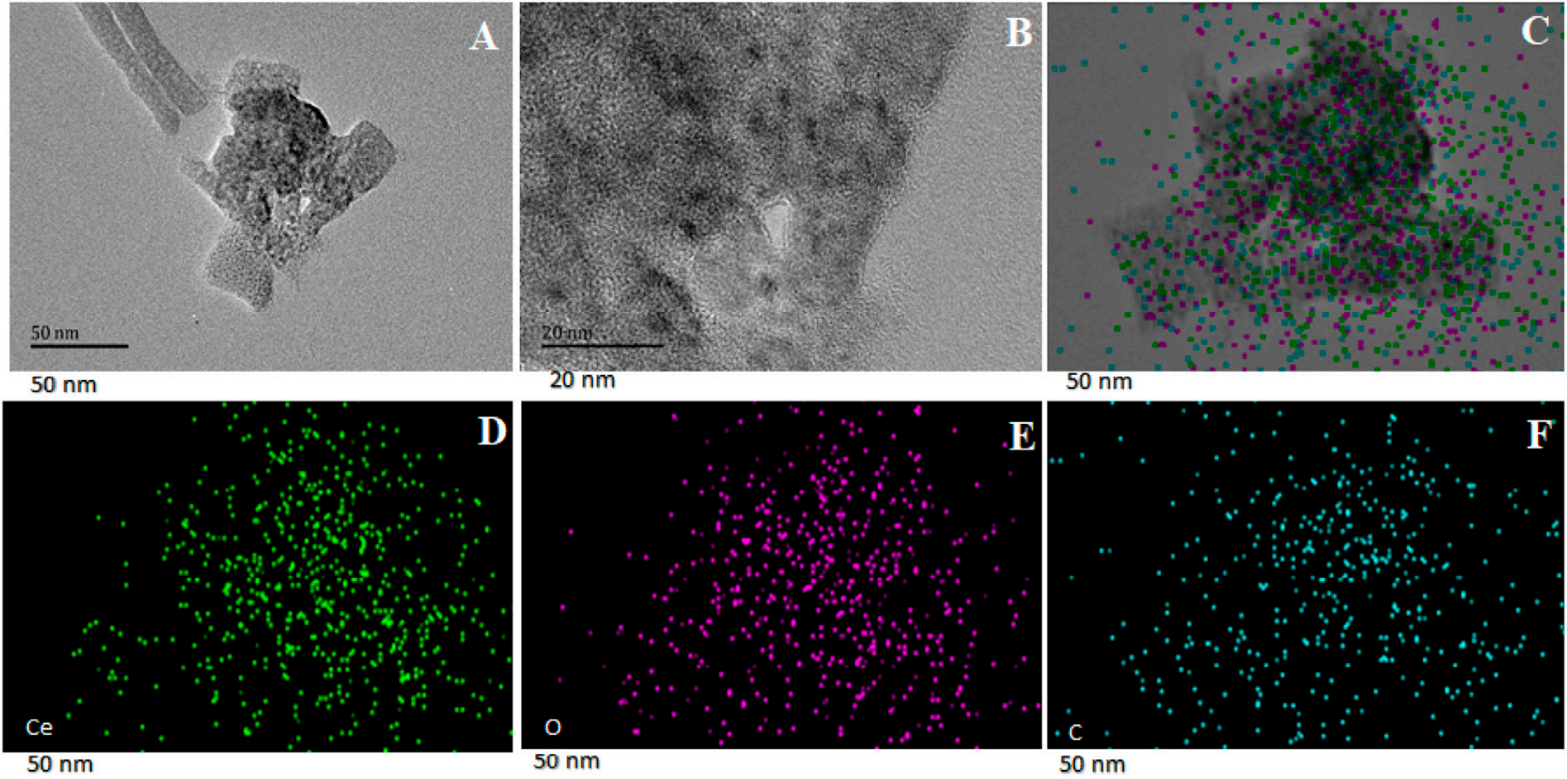
HRTEM images of Ce-MOF **(A,B)** and EDS elemental mapping **(C–F)**.

For Ce-MOF, the HRTEM images are displayed in Figures 4A,B, which reveal its rod-like morphology at different magnifications. These morphological findings are consistent with the SEM images presented previously. EDS elemental mapping of Ce-MOF is presented in Figure 4C. Different colors in the image indicate the presence of different elements, and all three colors are homogenously distributed among the whole structure. Ce-MOF contains cerium, oxygen, and carbon as its representative elements, and they are shown separately in Figures 4D–F. These results indicate that all three elements are present and evenly distributed, suggesting the successful formation of Ce-MOF.

**FIGURE 5:**
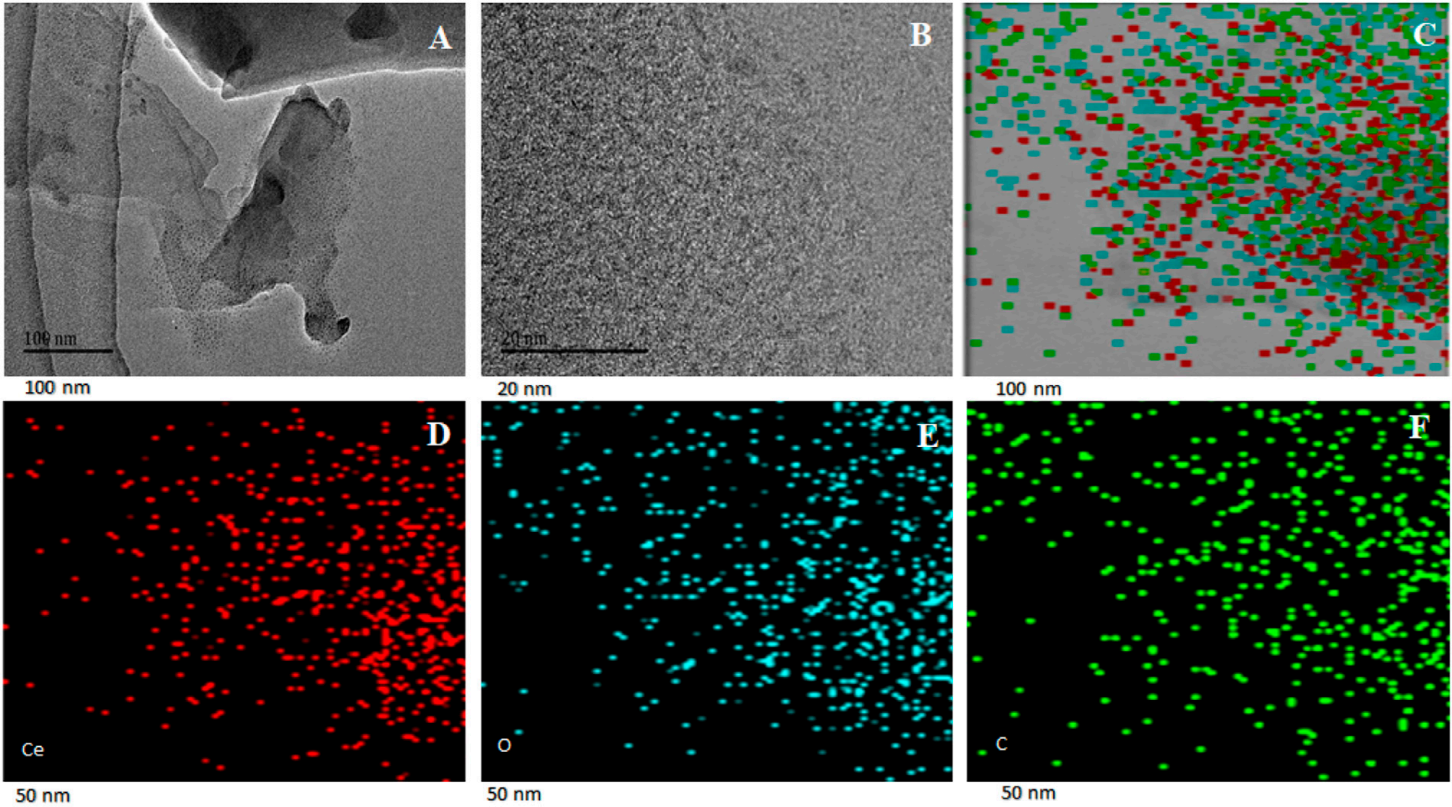
HRTEM images of GO@Ce-MOF composite **(A,B)** and EDS elemental mapping **(C–F)**.

HRTEM images and EDS elemental images for prepared GO@Ce-MOF composite are depicted in Figure 5. HRTEM images reveal slightly different morphology and layered structure, attributed to a higher carbon content after the composite formation with graphene oxide (Figures 5A,B). EDS elemental mapping of GO@Ce-MOF composite is shown in Figure 5C, indicating different colors representing different elements. The cerium, oxygen, and carbon content is shown by green, blue, and white colors in Figures 5D–F, respectively. The carbon content increases after composite formation.

#### BET

**FIGURE 6:**
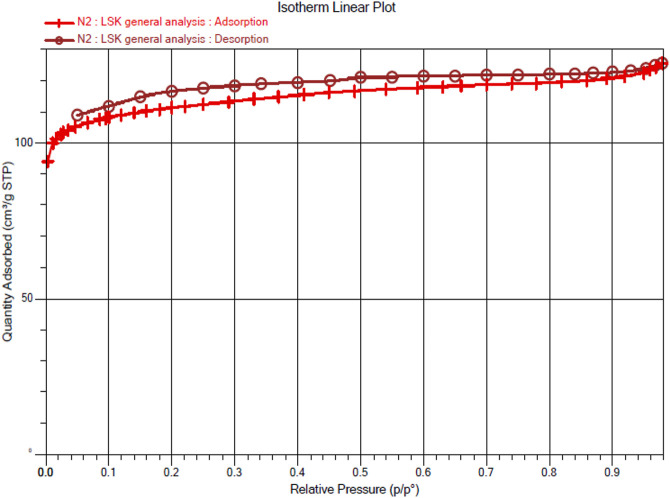
Nitrogen sorption isotherm of Ce-MOF.

**FIGURE 7:**
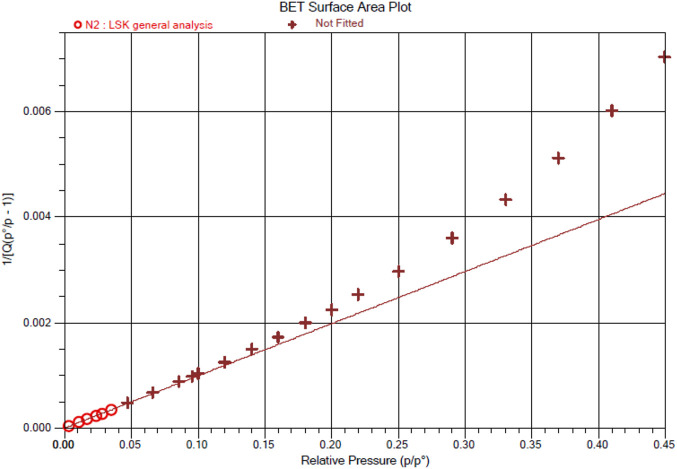
BET surface area plot of Ce-MOF representing surface area, slope: Y-intercept, and correlation coefficient.

Eternal porosity of Ce-MOF is validated at 77.086k by its N_2_ sorption isotherm. Ce-MOF was immersed in methanol (3-day) and finally in dichloromethane (3°days) and evacuated at an ambient temperature for 1°h to acquire activated samples before the measurement. The N_2_ sorption isotherm of MOF exhibits the characteristic type IV path that presents pore condensation correlated with characteristic adsorption–desorption hysteresis, as illustrated in Figure 6. These results illustrate the presence of mesopores in our Ce-MOF with the maximum N_2_ uptakes of 125.63°cm^3^/g (BET surface area of 439.8200 ± 0.3833°m^2^/g) with an adsorption average pore diameter (in term of pore size) and desorption average pore diameter (4V/A by BET) of 17,256° and 17.429°Å, respectively. The BET surface area plot of Ce-MOF representing surface area, slope: Y-intercept, and correlation coefficient: 439.8200 ± 0.3833°m^2^/g, 0.009894 ± 0.000009°g/cm³ STP, 0.000002 ± 0.000000°g/cm³ STP, and 0.9999985, respectively, is shown in Figure 7.

#### XPS

**FIGURE 8:**
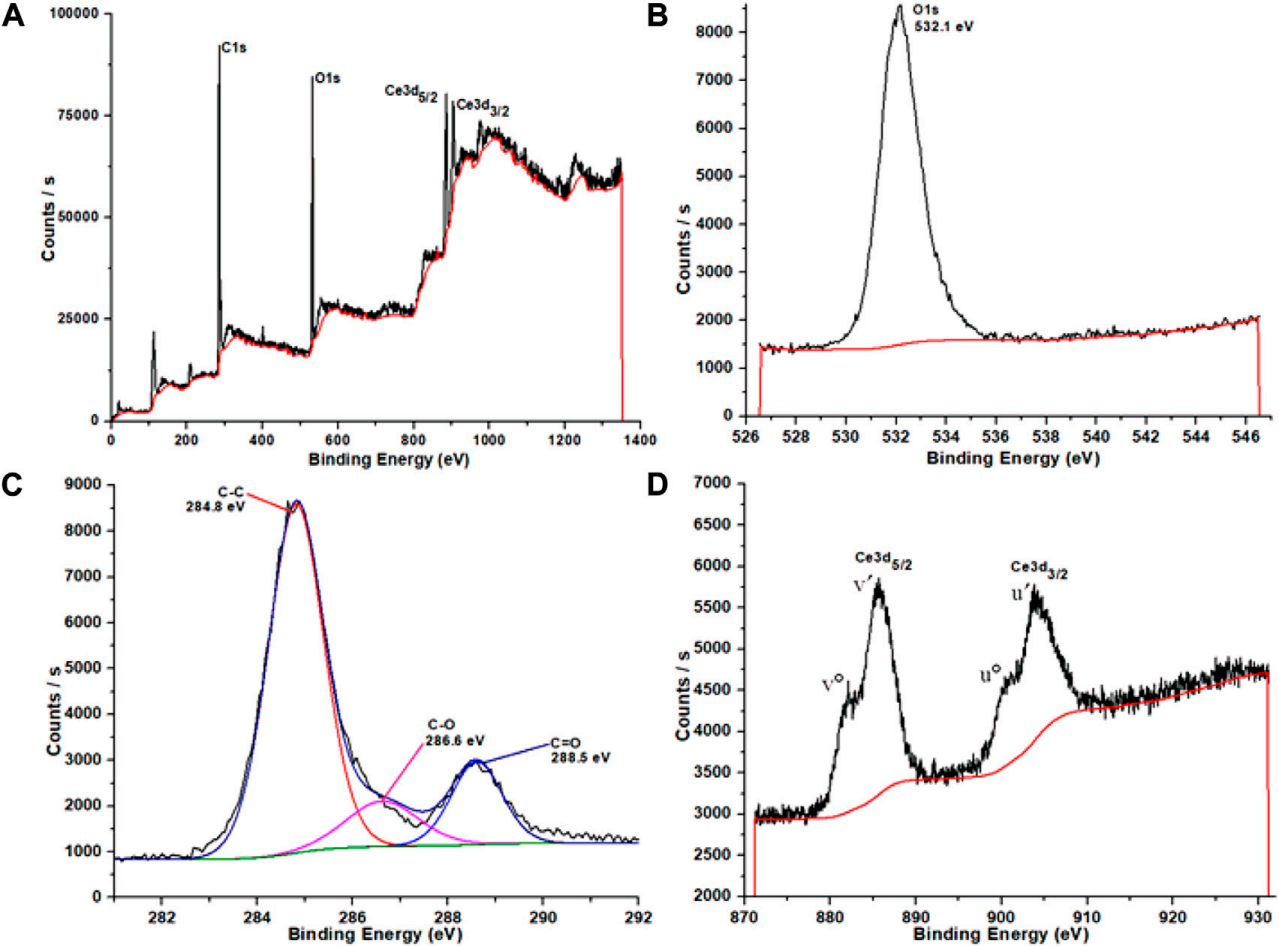
XPS survey spectra for Ce-MOF **(A)** and spectra of O1s **(B)**, C1s **(C)**, and Ce3d **(D)**.

X-ray photoelectron spectroscopy is carried out and is presented in Figure 8. The major peaks witnessed from the survey scan as C1s, O1s, Ce3d_5/2_, and Ce3d_3/2_ are centered at 284, 532, 885, and 903°eV, respectively (Figure 8A). O1s core level binding energy is noticed in the network at 532.1°eV that corresponds to organic oxygen found in the carboxyl group, that is, C = O (Figure 8B). In addition to the 284.8°eV peak (a symbolic peak for sp^2^-hybridized aromatic C = C carbons of the BTC linker), the C1s spectra show corresponding peaks at 285 and 289°eV for moieties retaining oxygen atoms in them (Figure 8C). Usually, for the BTC linker, C1s signals for -O-C = O and -C-O moieties appear at 288.5 and 286.6°eV, respectively. The aromatic peaks at 284.5°eV correspond to the C−C ring, while aromatic peaks at 285.2°eV correspond to the C = C ring. The high-resolution core-level spectrum for Ce 3*d* discloses the prevalence of Ce^3+^ species found in samples based on Ce-BTC. Core electron peaks for a couple of series of Ce 3d are shown, namely, Ce 3d5/2 and Ce 3d_3/2_ series accordingly (Figure 8D). These two peaks at 903.7°eV (3d_3/2_, u′) and 885.6°eV (3d_5/2_, v′) were caused by Ce^3+^3d-level characterized peaks. These two peaks belonged to Ce (III) (Peng et al., 2018) (Xiong et al., 2015) (He et al., 2020). The 3d_5/2_ peak appeared for Ce (III) at binding energies of 885.6 and 882.08°eV are labeled as v′ and v°, respectively. Additionally, for Ce 3d_3/2_, the binding energies witnessed at 903.7 and 901.0°eV are labeled as u′ and u°, respectively.

#### Electrochemical water splitting

**FIGURE 9:**
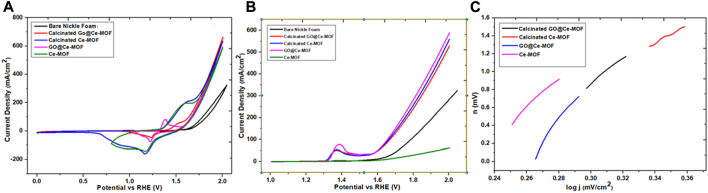
**(A)** CV, **(B)** LSV, and **(C)** Tafel slopes.

For the estimation of electrocatalytic performance of Ce-MOF, unadorned nickle foam (NF), composite of Ce-MOF (GO@Ce-MOF), calcinated Ce-MOF, and calcinated GO@Ce-MOF for water splitting, linear sweep voltammetry and cyclic voltammetry were performed. Figures 9A,B depicts the polarization loops in alkaline media (1°M KOH) deposited on the NF. Onset potentials (the minimum potential where the start of oxygen evolution reactions occurs) of 287, 275, 255, and 233°mV vs. RHE were noted for Ce-MOF, GO@Ce-MOF, calcinated Ce-MOF, and calcinated GO@Ce-MOF, respectively. Usually, a current density in the range of 10°mVcm^−2^ is taken as the point of reference for studying the intrinsic potential of any electrocatalyst employed in OER. The corresponding overpotential values noted at 10°mA/cm^2^ were 316, 311, 309, and 287°mV for Ce-MOF, GO@Ce-MOF, calcinated Ce-MOF, and calcinated GO@Ce-MOF, respectively. The obtained overpotential values indicated that calcinated GO@Ce-MOF possesses superior OER performance to Ce-MOF even at a significantly low onset potential. The presence of cerium in the MOF increases the cathodic selectivity of electrochemical water splitting. In the alkaline medium, a thin layer of cerium oxide or hydroxide is placed on to the electrode that ultimately enhances the electrocatalytic activity. Moreover, one of the most important parameters is the Tafel slope, representing the transport of electrons revealed at 96, 68, 58, and 39°mV/dec for Ce-MOF, GO@Ce-MOF, calcinated Ce-MOF, and calcinated GO@Ce-MOF, respectively, which demonstrates an excellent charge transfer inside the alkaline medium for our prepared and modified materials (Figure 9C). A smaller Tafel slope means that as the potential increases, the electrode resistance becomes small during polarization, and the EOR rate is fast. The Tafel slope of the GO@MOF catalyst is 58°mV/dec, which is lower than the Tafel slope of Ce-MOF (68°mV/dec). The Tafel curve is a portion of the polarization curves which is in a strongly polarized region. During polarization, improved potential and minor electrode resistance are predicted from a smaller value of the Tafel slope. The result suggests that incorporation of GO may enhance the catalyst surface area and facilitate the accessibility of the reactant to the electrode, thereby, capturing more ions and showing higher activity for water splitting. Additionally, these values ratify the faster reaction rate as well. Similarly, the small Tafel slope values in the case of calcinated GO@Ce-MOF further confirms lower onset and overpotential values which is a manifestation of propitious electrocatalytic performance of calcinated composite for OER. The current density of 589, 592, 634, and 661°mV/cm^2^ were noted for Ce-MOF, GO@Ce-MOF, calcinated Ce-MOF, and calcinated GO@Ce-MOF, respectively. It has been observed that the peak current density of GO@Ce-MOF is higher than that of simple Ce-MOF. The Go@Ce-MOF catalyst has an improved response than simple Ce-MOF. The results imply that the incorporation of GO in Ce-MOF increases the overall peak current density. This means that GO enhances the catalyst performance by increased surface area and conductivity of the catalyst. This high peak current density of GO@MOF suggested that the catalytic reaction requires smaller activation energy due to the presence of GO layers, which enhance the overall conductivity of the system. The enhanced electrocatalytic performance is most probably linked with the prevalence of new bare surface active catalytic sites inside calcinated Ce-MOF. The porous edifice of the presented material also contributed to an improved electron transfer.

By the equation TOF = I/4Q, the turnover frequency was calculated, where I denotes the current density at provided volts and Q denotes the Faradic charge for the aforementioned electrocatalysts that was below the voltametric peak and was further down with respect to the corrected baseline. The turnover frequency was calculated at 6.96°s^−1^ for prepared Ce-MOF, at 5.23°s^−1^ for GO@Ce-MOF, at 2.88°s^−1^ for calcinated Ce-MOF, and at 1.03°s^−1^ for calcinated GO@Ce-MOF. The TOF value shows an outstanding performance for the electrochemical transformation along with the great efficacy for our freshly prepared modified calcinated GO@Ce-MOF composite material.

**FIGURE 10:**
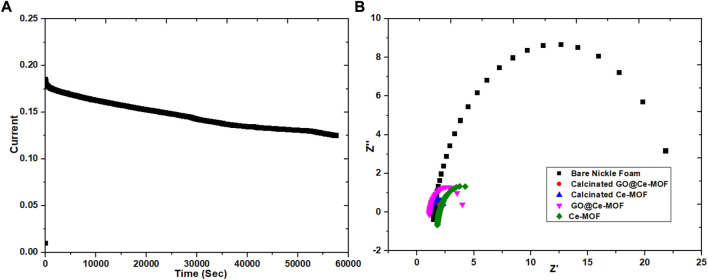
**(A)** Stability of calcinated Ce-MOF and **(B)** electrochemical impedance studies.

In addition to efficiency of the material, its stability on the relevant electrode surface is also counted as a crucial factor for estimating the electrochemical performance of the catalyst. Stability of the modified material is assessed by running 1,000 sequences along with chronoamperometry having a time duration of 15°h. Figure 10A illustrations revealed no obvious change from the 1st to 1000th cycle of cyclic voltammetry, which is a strong evidence for establishing extraordinary stability of the prepared calcinated GO@Ce-MOF composite edifice. The figure showing chronoamperometry of calcinated GO@Ce-MOF composite established in 15°h revealed indelible stability presented by the prepared material throughout the OER pathway. Conversely, preliminary diminution of current was transpired owing to oxygen bubbling on the electrode surface exteriorly.

**FIGURE 11:**
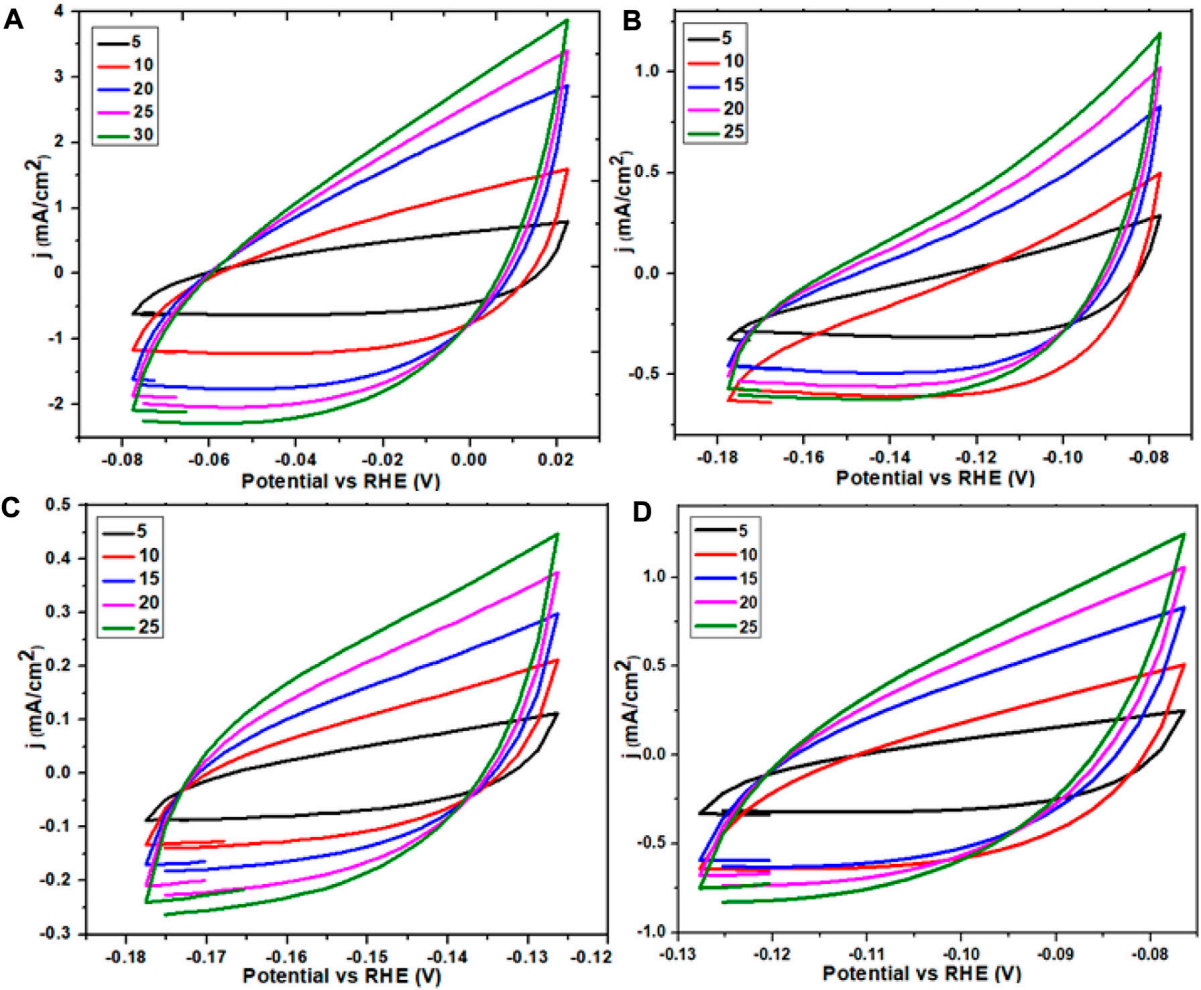
ECSA graph of Ce-MOF and straight line plot of scan rate vs. Δj **(A,B)** and ECSA graph of calcinated Ce-MOF and straight line plot of scan rate vs. Δj **(C,D)**.

Impedance measurements were also carried out to equally determine either the magnitude of conductivity or that of resistivity of the respective electrolyte and that of any sample materials (Figure 10B). In comparison to simple Ce-MOF, the catalytic materials, that is, GO@Ce-MOF, calcinated Ce-MOF, and calcinated GO@Ce-MOF show the lowest charge transfer resistance value. The charge transfer resistance of calcinated GO@Ce-MOF and calcinated Ce-MOF is significantly smaller than that of GO@Ce-MOF, which is further smaller than simple Ce-MOF, which means that the charge transfer of calcinated Ce-MOF and calcinated GO@Ce-MOF composite during the catalytic reaction is faster. Resistance of the catalytic material is described by the charge transfer resistance value inducted from an area of lower frequency and is denoted as R_ct_. On the other hand, for EIS measurements, R_s_ is symbolic for solution resistance, while R_ct_ and R_s_ are symbolic for the reaction kinetics carried out at the surface of electrode throughout OER accomplishment. For the prepared Ce-MOF, EIS shows a reduced Rs value (1.18Ω) and Rp value (3.04Ω), whereas for GO@Ce-MOF, the Rs value was 1.14Ω and Rp value was 2.9Ω. The Rs and Rp values for calcinated Ce-MOF were 1.11 and 1.33Ω, respectively. Similarly, the Rs and Rp values for calcinated GO@Ce-MOF were 1.9 and 1.21Ω, respectively, which indicated the higher conductivity of the material, facilitating the charge transfer reaction during electrochemical water splitting (Figure 11).

The electrochemical surface area measurement reveals the area of the working electrode by the accomplishment of the OER (oxygen evolution reaction). The working material’s activity could be enhanced by increasing the number of easily accessible active sites as well as by improving the electrochemical surface area. At a non-Faradic region, on behalf of variable scans, that is, 5, 10, 15, 20, 25, and 30°mV/s, several CV plots were collected. Exploiting the data of these plots, with the help of current density difference (j) at the anode and cathode, the Δj values were calculated. A straight-line graph was obtained by plotting the values of Δj against the aforementioned scan rates which revealed that the C_dl_ value is 8.1 for Ce-MOF, which was further divided with specific capacitance (0.040°mF/cm^2^) to obtain the ECSA value that was found to be 202 accordingly; for the composite of Ce-MOF (GO@Ce-MOF), if the C_dl_ value was 15.2, then C_dl_ values were divided with the aforementioned specific capacitance of 0.040°mF/cm^2^ to obtain the ECSA value of 380; for calcinated Ce-MOF, the C_dl_ value was 16.9, and by dividing C_dl_ values with the specific capacitance (0.040°mF/cm^2^), the ECSA value was 422; for calcinated GO@Ce-MOF, the C_dl_ value was 52, and by dividing C_dl_ values with the specific capacitance (0.040°mF/cm^2^), the electrochemical surface area was 1,290. The enhanced value obtained for the presented modified calcinated Ce-MOF (calcinated GO@Ce-MOF) material ascertains the accessibility and coverage of active sites that play a vital role in electrolysis regarding transfer of electrons.

## Conclusion

In summary, we successfully prepared Ce-MOF and GO@CeMOF by solvothermal methods and CeO_2_ from Ce-MOF and GO@CeMOF by calcination. The catalysts were investigated for their electrocatalytic activity for the oxygen evolution reaction (OER). The prepared electrocatalysts demonstrated exceptional results having low overpotentials, small Tafel slope, and low impedance with the highest peak current density. The overall electrocatalytic activity of the calcinated samples was higher than simple MOF and its composite. The accelerated catalytic activity of calcined samples (CeO_2_) as compared to Ce-MOF and GO@Ce-MOF can be attributed to the presence of more exposed active sites in the structure of CeO_2_. Meanwhile, high catalytic activity of GO@Ce-MOF, as compared to Ce-MOF, is due to the presence of GO layers, which enhance the overall conductivity of the system and provide more surface area with efficient active sites for the catalytic reaction. This study affords fresh insights into designing and engineering highly efficient electrocatalysts for water splitting and also opens up a new alleyway for conniving vastly ordered MOF-based surface dynamic materials for diversified electrochemical applications.

## Data Availability

The original contributions presented in the study are included in the article/Supplementary Material, further inquiries can be directed to the corresponding authors.
